
JOURNAL INFORMATION
==============================
NLM Title Abbreviation: Med Chir Rev
NLM Journal ID: 0034617

The Medico-Chirurgical Review

Publisher: Biomedical Journal Digitization Project

ARTICLE INFORMATION
==============================
PMCID: PMC4966182
PMID: 29917604
Article version: 1
Subjects: Reviews

Dr. Baron on the Origin and Progress of Tubercles

Print publication date: 1828 Jul 1
Volume: 9
Issue: 17
First page: 35
Last page: 41
1828 Apr 26
License: This work is free of known copyright restrictions. For more information, please see PMC Back Issue Digitization.
License URL: https://creativecommons.org/publicdomain/mark/1.0/

==============================

### IV.

*Delineations of the Origin and Progress of various Changes of Structure which occur in Man, and some of the inferior Animals; being a Continuation of Works already published on this Subject.* By John Baron, M.D. F.R.S. &c. &c. Quarto, with Plates. Longman's, 1828.

We have endeavoured to make the pathological investigations of Dr. Baron known as far as the circulation of this Journal extends; but we believe that his researches into changes of structure connected with hydatids and tubercles have not made much impression on his countrymen—much less so indeed than on the pathologists of other countries. We do not attribute this neglect to want of interest or importance in the subject—because pathology, in general, is not so much to the taste of our countrymen as it ought to be. When Dr. Baron hopes that the reader, in order to fully comprehend what he has here brought foward, will re-peruse his former volumes, he woefully miscalculates the taste of the day—the occupations of the profession—and the kind of literature which prevails. Dr. Baron has quarrelled most unmercifully with some of our continental brethren for mistaking certain opinions and expressions set forth in his former publications; but the fact is, that Dr. Baron's style is, in many places, very obscure, and we venture to assert, that not one half of his own countrymen have clearly understood him. Some allowance also should be made for the French physicians, few of whom are complete masters of the English language. If they have done Dr. Baron injustice in some things, they have done him more than justice in others. They have conferred on him a title which his own ungrateful country has hitherto withheld. He is much more frequently spoken of in the foreign Journals, as "Sir John," or "M. le Baron," than as plain Doctor.* This ought to have disarmed him of half his ire. This French politesse has been badly repaid by such hauteur or rather contempt, as the following mode of speech indicates. "A writer, who signs himself Meriadec Laennec," &c. Dr. Baron will not enhance his own dignity by this mode of address to respectable foreigners who take notice of his works. All this ire has arisen from Monsieur Laennec mistaking Dr. Baron on the subject of generation of hydatids. Dr. B. observed that the same state of the system which calls into existence a solitary tubercle, may *generate* an indefinite number. The Frenchman thought that it was from the original or mother hydatid, that the future generations sprang—and thus Dr. B. was mis-

* Dr. Wilson Philip is very generally styled "Sir W. Philip," both on the Continent and in America. We sincerely hope that these complimentary mistakes of our polite neighbours, may soon be converted into realities.

understood on (what he considers) a most important point of pathology. It is clear that Dr. Baron is well aware of the animalcular nature of hydatids; but his object is not to explain the origin of these bodies, so much as to trace their change into a tubercular state, which constitutes the progress of the disease he has been so long investigating.

"Admitting for the sake of argument 'that hydatids are animalcules, it has, I trust, been shown that it is to the loss of the hydatidal character altogether and the transformation of these bodies that the morbid appearances in this and many other diseases are to be referred."

The doctor's design is, to show that such transformations do take place, and occasion a great variety of disorganizations in the animal frame. This doctrine, he conceives, has nothing to do with speculations respecting the origin and vitality of hydatids. We agree with Dr. Baron that the pathological questions at issue cannot be properly determined by examining disorganizations in their last stages, when all the elementary characters are lost. It is by tracing them through all their progressive changes that satisfactory information can be obtained. It is obvious that this can only be done (except on very rare occasions) on inferior animals. The inquiry, in this way, can happily be prosecuted as well in this country as on the Continent, where human pathology has greater advantages.

We think Dr. Baron has occasioned a great waste of useless learning in his attempts to trace auscultation and percussion to Hippocrates. No one can fail to see that Dr. Baron is less actuated by a desire to promote these aids to diagnosis, than to prove that the discovery was not French—Dr. B. being an antigallican worthy of the days of Pitt. When will science divest itself of these little narrow prejudices, and look to truth as the only object of pursuit, without any regard to the soil which produces it?

In the second chapter, our author comes immediately to the real business of the book. The lungs, he observes, from the peculiarity of their structure, do not offer so good a field for tracing the disorganizations in question as the liver. In this last, the slightest deviation from the natural condition is easily discernible, and the contrast between the unsound and healthy part is very manifest. It is, also, an organ which is more frequently diseased in the inferior animals than almost any other viscus.

"There are two ways in which the first indications of the class of diseases now under consideration become visible. The one is denoted by the enlargement or greater development of the lymphatics; the other, by the formation of little vesicles. Both these, for the most part, occur together in the same organ; but it is sometimes otherwise.

"To illustrate this matter a little more fully, I shall state what any one may witness who will examine the first appearances of the changes in question. Let us take for example a liver that is becoming diseased. The first thing that strikes the eye is a manifest enlargement of the lymphatics. They are quite apparent, and evidently occupy a larger proportion of the organ than they do in their natural state. At this time they may retain their transparency; but generally they soon become opake, and white, or of a pearl colour. A section of the organ at this period gives a mottled appearance not unlike that which is exhibited by the cut surface of a nutmeg. As the disease advances, a greater disproportion is exhibited between the diseased and sound parts. The white portion very much predominates. Ultimately, the brown or interstitial parts, which are remains of the proper glandular texture are altogether removed, and an apparently scirrhous mass is left in its stead. The different steps in this progress will be more clearly demonstrated when I come to the description of the plates. This may be considered as one of the most frequent and simple forms in which disorganization takes place. It is very often found in this state in one viscus, or even in one portion of a viscus; while other parts exhibit it in combination with other changes, some of which I am now to notice.

"These consist in the formation of vesicles, and tubercles, and tumours. The first step in this series of disorganizations may be accomplished in one of two ways. One is, the expansion of the lymphatics at the point of intersection where different branches meet. This variety, of course, occurs most frequently in the substance of the viscera, where the distribution of the lymphatics is different from what it is on their surface. The other occurs when the vesicle is formed immediately in the course of the lymphatic trunk. In the first mentioned case the vesicle is not quite so regular in its shape as it is in the last; and as it advances, and becomes consolidated into a tubercle, there is in some instances a sort of contraction which gives the surrounding part a radiated, puckered, appearance: all the lymphatics which are connected with the tubercle being thickened and changed."

Dr. B. then dwells somewhat minutely on the course or progress of tuberculation. In the first instance, a small vesicle is formed. It may go on advancing in size, and retaining its transparency, not materially interfering with the functions of the organ. Supposing the liver still to be the scene of action, the following phenomena will present themselves to the attentive pathologist.

"To illustrate my meaning, let the reader suppose, what very often occurs, that a vesicular body of this kind is generated a little way below the outer surface of that organ. It increases in size in every direction, and, at length, a small portion of its circumference is seen like a transparent speck amid the dark surrounding texture. This part becomes more visible as the body grows; and, as its magnitude increases, the manner in which it dispossesses the true texture of the part, where it is formed, is beautifully seen. In the liver, where the glandular texture is divided and subdivided, these partitions may be perceived gradually expanding like a thin film, or curtain, over the increasing growth of the vesicle. After a time they are completely absorbed, and the boundaries of the disorganization become clearly defined.

"When the first small deviation from the healthy state shows itself, it is not accompanied by change either in the colour, or texture of the adjacent parts. They retain their original properties, and it is not till the morbid part undergoes its other changes, and assumes consequence either from its increasing bulk or from the interruption that it may cause to the functions of the organ, that any material alteration in the contiguous structure is perceptible."

The inferior animals will furnish any one with these illustrations—but to those who will not take the trouble of investigating nature, Dr. Baron's plates present an excellent succedaneum.

"In the lungs, the primary condition of the disorganizations is not less illustrative of the above description. The clear and untransformed vesicles may be seen here and there interspersed through its substance, or studded over its surface. In this state, unless they happen to be formed in great numbers, and at no great distance from each other, they cause but little interruption to the functions of the part; but things do not remain long in this condition. A change both in the diseased structure, and the portion of the lung connected with it, soon takes place; and, subsequently, all those progressive alterations occur which I have elsewhere endeavoured to describe."

Before entering on a description of the plates, Dr. B. details the results of a few experiments on rabbits—which are, however, only a repetition of those detailed in his former work, as performed by Dr. Jenner. That gentleman found that it was possible, by feeding animals on unwholesome food, to call up diseases in different organs, especially in the liver. He ascertained also that, by altering the external circumstances, and improving the diet, those disorganizations, which had advanced to a considerable extent, might be removed.

"I had it not in my power, on a former occasion, to describe the different steps in the progress either towards disease or a restoration of health, because I was not furnished by Dr. Jenner with any thing farther than a statement of the general result of his observations. I therefore deemed it proper to bring the subject more immediately under my own eye, and to watch the various stages in the processes to which I have referred. For this purpose I placed, about the end of last April (1825), a family of young rabbits in a confined situation, and fed them with coarse green food such as cabbage, grass, &c. They were perfectly healthy when put up: on the third of June one of them died. On examining its liver, the primary steps in the disorganizations, which I am now considering, were evinced in a very manifest and interesting manner. The first thing that showed itself was a number of transparent vesicles about the size of a pin's head studded over its external surface; some few of them had attained a larger size and might equal the tenth of an inch in diameter. With these appearances there was an increase in the magnitude of all the lymphatics of the organ. This was visible in the superficial ones, as well as in those which belonged more particularly to the proper texture of the organ: and it was chiefly at the points of intersection of the latter that the transparent vesicles, above described, were seen. These appearances were also visible on cutting into the organ, but it did not exhibit any other mark of disease.

"On the 12th of June another rabbit died: in it the disease was considerably farther advanced. The whole liver appeared of a lighter colour, and was somewhat enlarged. It was studded with tubercles; some of them semi-transparent, others quite opake, and nearly firm in their texture, and of a bright yellow colour. A corresponding increase in the disease of the lymphatic vessels was also apparent; that is to say, they were larger than in the last case, and had lost their transparency; and there was, of course, a corresponding diminution of the natural hepatic texture.

"June 16th. A third rabbit died this day. The progress of the disease was very clearly marked. One of the lobes was universally pervaded by small tubercles about the size of millet seed: they stood prominent on the outer surface so as to give it a granulated appearance. They were developed in such numbers as almost entirely to have taken place of the true hepatic structure; and to leave a mass of irregular surface and of a straw or ash colour in its stead. Attached to the lower surface of the liver there was an hydatid about the size of an hazel nut.

"June 18th. This day a fourth rabbit died: a considerable number of hydatids were attached to the lower surface of the liver, and lay between it and the smaller curvature of the stomach. They were not quite globular, and were becoming opake; and their contents in colour and consistence looked like calves'-foot jelly. There were six or eight similar, but smaller and more transparent bodies attached to the omentum.

"The liver itself contained many tubercles, but fewer than in the last case. They had been developed at greater distances from each other; and had consequently acquired a greater size. The other parts of the viscus were apparently sound.

"At this time I removed three young rabbits from the place where their companions had died to another situation. They had all the external signs of being diseased like those already described. They were remarkably low in flesh, whilst the abdomen was tumid, and the whole skin scaly and unhealthy. Their new abode was dry, and kept clean; and they were fed chiefly upon bran and oats, with a moderate proportion of clover and dandelion.

"About ten days after this change had taken place I killed one of them. The animal had increased a little in flesh, but the other external signs of disease existed, and the progress of the internal disorganization had not been arrested. The liver was very much enlarged, and there was a greater development of the whole of the lymphatic system of the organ than in any other of the former instances. There were also a very great number of the tubercles of different sizes and in different stages of their progress, so that scarcely any part of the liver remained in a sound state. In Plate III. Fig. 2, will be found a representation of this diseased part. Any one accustomed to examine appearances of this kind will perceive how formidable the disorganization is. I present it to the reader in order to show how far change may take place, and yet the healthy condition of the part be restored. In order to put this point to the test I killed the fellow of this last-mentioned rabbit on the 5th of August. It had been fed exactly as I have already described. It was very much improved in health, and on examining the liver it was found comparatively in a sound state. Its colour was natural, it was not much enlarged, and there were only a very few tubercles discoverable in it."

These facts, so far as they go, are very interesting, both as illustrating the origin and progress of disease, and in furnishing therapeutical hints that ought not to be overlooked.

The zealous author next proceeds to the detail of some facts drawn from the examination of several other animals, in which, disorganizations akin to those described, are frequently met with.

"On the 2d of April 1823 I had a horse killed which had laboured under glanders for nearly two years: most of the internal organs, but especially the lungs, liver, and spleen, indicated the existence of tuberculous disease: the former in particular evinced its ravages. Large portions of that viscus containing solid masses formed, some by the aggregation of tubercles of different sizes, and others which presented a bright yellow, firm texture without signs of division of parts, except when, here and there, a well-defined circular body interrupted the uniformity of its appearance. It is extremely likely, for reasons which I have elsewhere stated, as well as from the appearance of other portions of this disease, that an examination of it at an earlier period would have shown the boundaries of its elementary parts in a much more distinct manner.

"A considerable portion of these diseased masses was in a state of ulceration, and had large and direct communications with the broncial tubes. Corresponding diseased appearances were also found in the liver and the spleen. It is besides proper to mention that the mesocolon was thickened, and contained tubercles; and that the mesenteric glands were diseased and enlarged."

Dr. B. opened a sheep where tuberculous disease was advancing. Both lungs and liver shewed the progress of the disorganization in a very clear manner. He also opened another glandered horse, when the disease had been of much shorter duration than in the former. The early stage of disorganization was, therefore, more conspicuous. The lungs, at first sight, appeared healthy. But, on closer examination, a number of circular vesicles were seen—some transparent—others opake or yellow. Some presented an oblong shape, and seemed clearly to have been formed in the course of the transparent vessels, which Dr. B. conceived to be lymphatics. There was not the slightest mark of increased vascularity in connexion with these disorganizations. Blood-vessels were seen traversing the lungs, as in a healthy state. Subsequently Dr. B. traced the same phenomena in other animals, especially in hogs. In all these examples, it was found that, except the increased magnitude of the lymphatics, and the occasional appearance of the vesicle, there was no indication of any other change. This, Dr. B. thinks, will apply to the early state of every variety in this genus of disorganizations:—"thus demonstrating that diseases which we seldom or never see in the human subject, till they have entirely destroyed the texture of the parts they attack, take their rise from beginnings so small, and apparently unimportant, as scarcely to attract attention." In the second plate, Dr. Baron presents us with portions of the lung and liver of the same sheep. In each, the disease, in its early, as well as in its more advanced stage, is seen. The similarity in the progress, both as regards the disorganization itself and the state of the surrounding parts, is very apparent.

Dr. Baron next draws our attention to the origin and growth of tubercles and tumours in the uterus and its appendages. There is no disorganization of this genus more common than what is denominated ovarian—though the disease is very often unconnected with the ovaries themselves.

"In general the following is the progress discernible in this variety of disease. One or more transparent vesicles may be seen attached to the Fallopian tubes, or to any portion of the uterus or the parts contiguous. These as they increase in size undergo various changes, and at last a compound disorganization is formed, exhibiting different properties according to the time at which it may have been examined, and the actual state of its elementary parts. Two cases which I have lately examined are explanatory of this statement. In one the disorganization was in its primary state. There were distinct pellucid vesicles containing a transparent fluid, the smallest of which was not much larger than the head of a pin; the largest was about the size of an hazel nut. Some slight change had taken place in the coats of the latter, they having become, in some degree, dense and opake. In the other case, of which a representation will be found in Plate III., a more advanced state of the same variety of disease may be seen. Clusters of vesicles, and tumours were found hanging from each Fallopian tube; and the progress in the transformation was demonstrable in the most satisfactory manner. One large vesicle of about an inch in diameter was quite pellucid; another, about the same size on the opposite side, was half pellucid, and half opake and solid: the progress from the soft transparent state towards consolidation being thus clearly discernible. For a farther elucidation of this case I would refer to the Plate. It will be observed that the tumours hang downwards. This is not the position in which we generally find them in their last stages. The reason of this is obvious. If they increase much in size, their downward growth is necessarily prevented by the resistance which they meet with from the pelvis: they are, of course, forced to take another direction, and ascend into the abdomen, where they often acquire an enormous magnitude. For descriptions of the appearances of the disease, and for an account of the manner in which these appearances are formed, I must refer the reader to the different parts of my Inquiry, where I have described such disorganizations; and for proofs that these descriptions are faithful, the reader may examine the plates recently published by Mr. Lizars."

When tubercles are developed on the peritoneal surfaces, constituting ultimately that painful disease described in our author's former work (tuberculated accretion of the serous membranes) they may be seen, in their early stage, standing prominent and apart from each other, on the surface of the intestines, the colour and structure of the surrounding parts having undergone no change. But as they grow in size and number, other deviations become manifest. An increased vascularity may be seen; and with it a thickening and roughness of the peritoneal surfaces—and ultimately, the formation of those dense accretions, which often unite the whole of the abdominal viscera together. What are the obvious practical lessons deducible from these pathological enquiries? We shall let the author speak for himself.

"What boots it to be told when we detect an enlargement of the liver, or a tumour in the abdomen, or tubercles in the lungs, that inflammation acute or chronic has been doing its work? Did the practice founded upon this dictum ever enable us to overcome the disease? I believe that except under some rare and peculiar circumstances it never did. There is no good reason for believing, as I have often already stated, that inflammation causes, or even accompanies the disorganizations of which I have been speaking in their early stages. It may, and does sometimes, come on when they are advancing to their terminations, but even then it is by no means a necessary accompaniment, and is manifestly excited by the previous disease.

"What on the other hand, it may be asked, have been the means by which a constitution indicating the commencement or progress of these disorganizations, has been improved, and the threatened evils averted? By what agents have we seen tumours and morbid growths removed? Have the remedies that usually subdue inflammation been sufficient for this, or have not other agents been necessary? The experience of every man will answer. It is much to be lamented that our resources in this class of diseases are as yet so limited. If, however, their nature be such as it appears to be, it cannot be doubted that agents capable of controlling their progress may ultimately be acquired. Indeed I am convinced that remedies of that sort are already in our hands. Such remedies have removed many disorganizations that formerly would have resisted all known means; and there can be no doubt that their employment, in the early stages, promises results still more favourable. Unfortunately, the great difficulty lies in detecting the disease before it has assumed a fixed and formidable character. But that difficulty is not insurmountable. It may in part be obviated, first, by detecting the earliest morbid appearances; and, then, by associating them with the symptoms which they produce."

Dr. Baron, no doubt, alludes to mercury, iodine, and some other medicinal agents in the removal of morbid growths. The doctrine of Dr. Baron is certainly very important, in a practical point of view, especially as regards that dreadful scourge of the human race—tubercular phthisis. Laennec is evidently of the same opinion as our author, in respect to the non-inflammatory nature of tubercles in the lungs. Experience also shews that the disease is not to be cured by depletion and low living. Tubercles in the lungs, as in every part of the body, produce, occasionally, inflammation and disorganization of the parts contiguous; but this is a very different thing from the production and growth of the tubercles themselves. In such cases, we must bleed locally to relieve the suffering sound parts, while we give tonics and nourishing diet to check the morbid process of tuberculation.

The description of four plates containing a great number of figures most beautifully drawn and coloured, conclude this fasciculus. We have endeavoured to convey as clear an idea, as possible, of the matter contained in the letter-press of the work; but Dr. Baron is a hard man to please; and we should not be much surprised if he complains that we have misunderstood him. We hope, however, to escape with more whole bones than "one who signs himself Meriadec Laennec," appears to have done.
